# Collagen density modulates triple-negative breast cancer cell metabolism through adhesion-mediated contractility

**DOI:** 10.1038/s41598-018-35381-9

**Published:** 2018-11-20

**Authors:** Emma J. Mah, Austin E. Y. T. Lefebvre, Gabrielle E. McGahey, Albert F. Yee, Michelle A. Digman

**Affiliations:** 1 Department of Chemical Engineering and Materials Science, University of California, Irvine, Irvine, California USA; 2 Department of Biomedical Engineering, University of California, Irvine, Irvine, California USA; 3 Laboratory for Fluorescence Dynamics, University of California, Irvine, Irvine, USA

## Abstract

Extracellular matrix (ECM) mechanical properties upregulate cancer invasion, cell contractility, and focal adhesion formation. Alteration in energy metabolism is a known characteristic of cancer cells (i.e., Warburg effect) and modulates cell invasion. There is little evidence to show if collagen density can alter cancer cell metabolism. We investigated changes in energy metabolism due to collagen density in five breast cell lines by measuring the fluorescence lifetime of NADH. We found that only triple-negative breast cancer cells, MDA-MB231 and MDA-MB468 cells, had an increased population of bound NADH, indicating an oxidative phosphorylation (OXPHOS) signature, as collagen density decreased. When inhibiting ROCK and cell contractility, MDA-MB231 cells on glass shifted from glycolysis (GLY) to OXPHOS, confirming the intricate relationship between mechanosensing and metabolism. MCF10A cells showed less significant changes in metabolism, shifting towards GLY as collagen density decreased. The MCF-7 and T-47D, less invasive breast cancer cells, compared to the MDA-MB231 and MDA-MB468 cells, showed no changes regardless of substrate. In addition, OXPHOS or GLY inhibitors in MDA-MB231 cells showed dramatic shifts from OXPHOS to GLY or *vice versa*. These results provide an important link between cellular metabolism, contractility, and collagen density in human breast cancer.

## Introduction

Cancer cells can modulate their energy metabolism to meet nutritional, biosynthesis and respiration requirements for maintaining malignancy. One of these factors is the metabolic state of the cancer cell due to their tendency to undergo aerobic glycolysis, known as the Warburg Effect^1–3^. Although it produces less ATP per molecule of glucose, glycolysis (GLY) is a more rapid way of producing ATP and is able to meet the high demands of energy to fuel processes such as invasion, migration, and matrix degradation^4–7^. Along with high turnover of ATP production, a byproduct of lactic acid and high acidification also has been shown to benefit cancer cell survival and upregulate invasiveness^8^.

Mechanical properties of the extracellular matrix (ECM) is also a known factor that regulates cell migration and cancer invasion^9–13^. Cells interact with the surrounding ECM through integrin-mediated adhesions and focal adhesions (FAs), that are clusters of over 150 proteins^14,15^. These complexes tether to the cell’s mechanosensing network through actin filaments and regulate processes such as adhesion, migration, and proliferation^16–19^. Recent studies have shown that integrin-mediated adhesions interact with the metabolic pathway of the cell through the PI3K/AKT/mTOR pathway and that this could be a potential method of switching the Warburg effect^20–22^. Many of these studies use biochemical assays which are invasive and often lose information which exists in live cell samples. In our approach, we used a non-invasive fluorescence imaging technique to measure ECM density, study live cell behavior, and map energy metabolism within each cell.

Fluorescence lifetime imaging microscopy (FLIM) has been shown to be a powerful technique to measure metabolic indices of live-cells^23–29^. By looking at the fluorescence lifetime of nicotinamide adenine dinucleotide (NADH), a metabolite involved in OXPHOS and GLY, we can determine the population of free and bound NADH due to their difference in lifetime decay. This will allow us to quantify the “metabolic trajectory”, known as the “M trajectory”, of the cell at every pixel of our image and determine if the cell is undergoing OXPHOS or GLY^28^. This trajectory has also been shown to correlate with results found in conventional biochemical assays when OXPHOS or GLY inhibitors are used to shift metabolic signatures towards one another^27,28,30^. The advantage of this imaging technique is that it is non-invasive and is able to image real-time changes in metabolism.

For this study, we measured free and bound populations of NADH within different cancer cell lines and a non-tumorigenic cell line when seeded on collagen substrates of different concentrations (1.2 mg/mL and 3.0 mg/mL) and on glass. The microstructural properties of this substrate, including collagen density and fiber diameter, were measured using image correlation spectroscopy^31^. The 3.0 mg/mL and 1.2 mg/mL collagen substrate had collagen fibers of similar size, but the 3.0 mg/mL collagen substrate gave rise to a denser ECM than the 1.2 mg/mL substrate by 3X. The 3.0 mg/mL collagen substrate was also determined by rheology to be one order of magnitude stiffer than the 1.2 mg/mL collagen substrate. In this paper, we will refer to the density of the collagen as our means to distinguish the difference between the two ECM substrates. We are also aware that each cell line requires different media conditions for culturing and these affect the metabolism. However, we are interested in the effects of collagen density on each cell line independently. The highly metastatic triple-negative breast cancer cell (TNBC) lines, MDA-MB231 and MDA-MB468 were grown on collagen and glass substrate to observe their metabolic shifts. MDA-MB231 showed a shift towards a more glycolytic signature as collagen density increased. Inhibition of cell contractility with the addition of Y-27632 shifted all the cells on all substrates to a more OXPHOS signature compared to their uninhibited controls. This further shows that integrin-mediated adhesions behave as mechanosensors and these adhesions can alter metabolism. MDA-MB468 cells did not show changes in response to collagen density, however, the cells showed increase OXPHOS on both collagen substrates when compared to glass. MCF-7 and T-47D, the less invasive breast cancer cells, were also tested and did not show changes in metabolic signatures.

Non-tumorigenic breast cell line MCF10A showed slight changes in NADH free:bound ratio only on the low-density collagen surface, indicating that this property is more prominent in MDA-MB231 and MDA-MB468 cell lines. Other cancer cell types, U251MG glioma and A375MM melanoma cell lines, were evaluated under the same conditions. The U251MG cells had no significant differences in their NADH free:bound ratio in response to collagen density. A375MM cells did not adhere well to the collagen substrates could be a reason for their lack of change in their NADH free:bound ratio, indicating that the mechanosensing network must be established in order to undergo metabolic reprogramming. Inhibition of OXPHOS or GLY in MDA-MB231 cells showed shifts in NADH free:bound ratio with respect to each treatment towards their metabolic counterparts across all surfaces, and further confirmed that it is indeed the metabolism that is being altered by the ECM. MCF10A cells showed a shift when OXPHOS was inhibited only on our denser collagen substrate and on glass when GLY was inhibited. The results found in our work here show that both the mechanosensing and metabolism pathways are interconnected and can be modulated through ECM mechanical properties. This will provide further information to develop cancer therapies which target either or both of these pathways to decrease cancer cell invasion.

## Results

### Collagen characterization measurements

**Figure 1:**
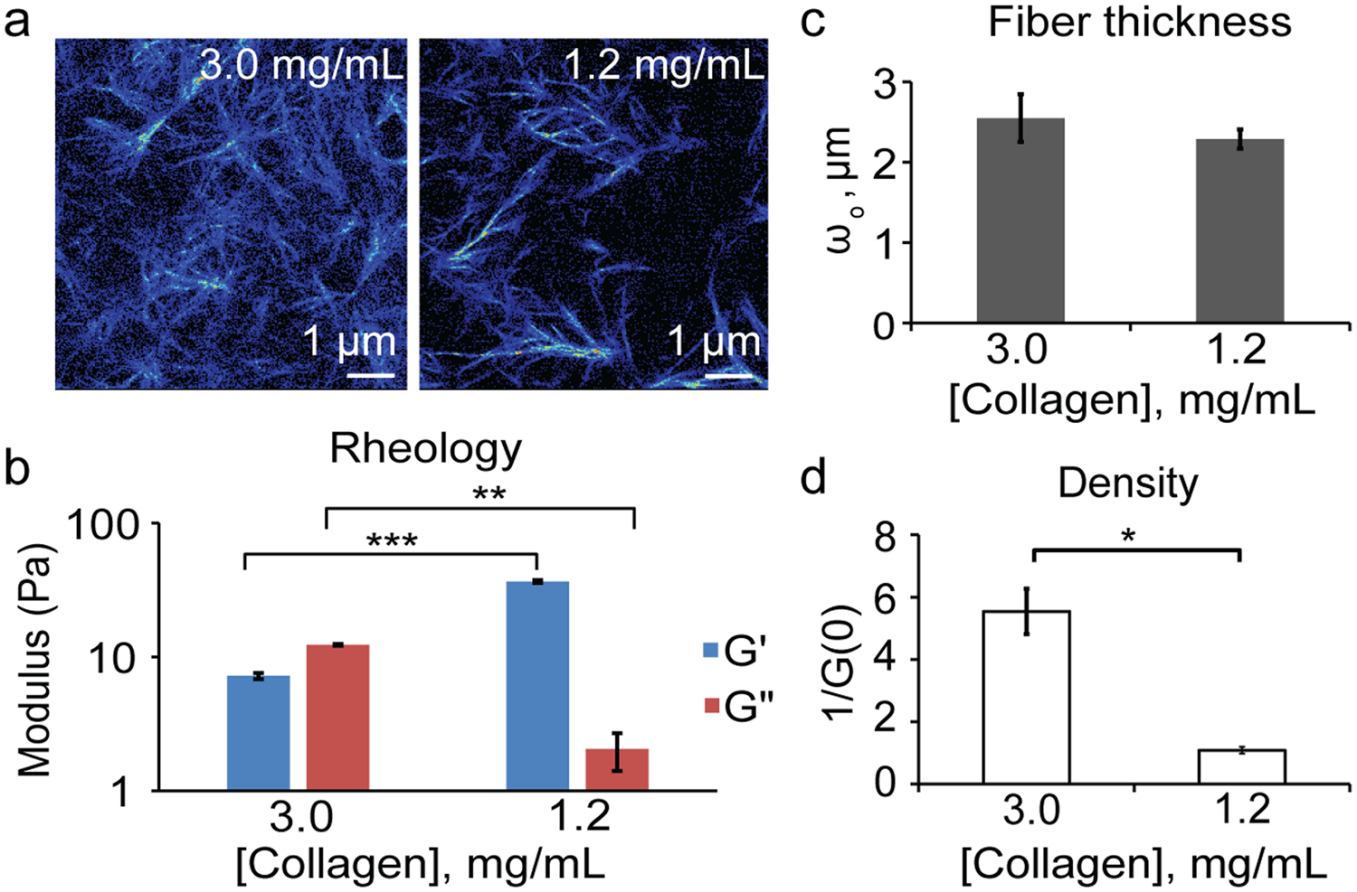
Quantification of collagen substrates. (**a**) Second harmonic generation images of 3.0 mg/mL (n = 9) and 1.2 mg/mL collagen substrates (n = 7); where n = total number of collagen samples measured. (**b**) Modulus of collagen substrates at 10% strain and 1 Hz. (**c**) Quantification of fiber size (ω_o_) and (**d**) density of collagen substrates. *p < 1e-3, **p < 1e-26, ***p < 1e-57.

Tilghman *et al*. postulated that cellular metabolism can be altered when MDA-MB231 cells are cultured on soft (300 Pa) versus stiff (19200 Pa) matrices due to the fact that cells stayed in the G1 phase cell cycle phase longer^32^. Indeed, their results using cell lysates with ATPlite assay and protein synthesis assays confirmed their hypothesis. The substrates used in those experiments were limited to polyacrylamide gels that have a large rigidity/flexibility range, but it is not physiological. In our approach, we used collagen monolayers prepared at two different concentrations of 1.2 mg/mL and 3.0 mg/mL. Second harmonic generation (SHG) images were taken to measure the fiber thickness, and density was measured using image correlation spectroscopy (ICS). Previously in our lab, we have shown that the mechanical properties of collagen obtained through SHG and ICS correlated to those obtained by rheology or scanning electron microscopy images^31,33^. For this analysis, the ω_o_ value gives the waist of the auto-correlation function and based on the size of the point spread function of the laser (~0.3 µm at the waist). A larger ω_o_ indicates thicker fibers. 1/G(0) quantified the density of the matrix which is the height of the auto-correlation function extrapolated from the first measured point. A smaller 1/G(0) value corresponds to denser matrices. 3.0 mg/mL and 1.2 mg/mL collagen substrates showed similar average values of ω_o_ of 2.55 and 2.29 (Fig. 1c). However, the 3.0 mg/mL collagen has a significantly larger average value of 1/G(0) of 5.54 compared to that of the 1.2 mg/mL collagen at 1.09 (Fig. 1d). This confirms that the 3.0 mg/mL collagen substrates have a denser network of collagen although their fiber thicknesses are similar. Rheology measurements were also done to quantify the modulus of the substrates. 3.0 mg/mL and 1.2 mg/mL collagen substrates were measured and have averages of 38.12 Pa and 5.66 Pa, respectively (Fig. 1b), showing that the 3.0 mg/mL collagen substrates are about one order of magnitude stiffer than the 1.2 mg/mL substrate.

### Triple-negative breast cancer cells shifts towards glycolytic signatures on denser collagen substrates

**Figure 2:**
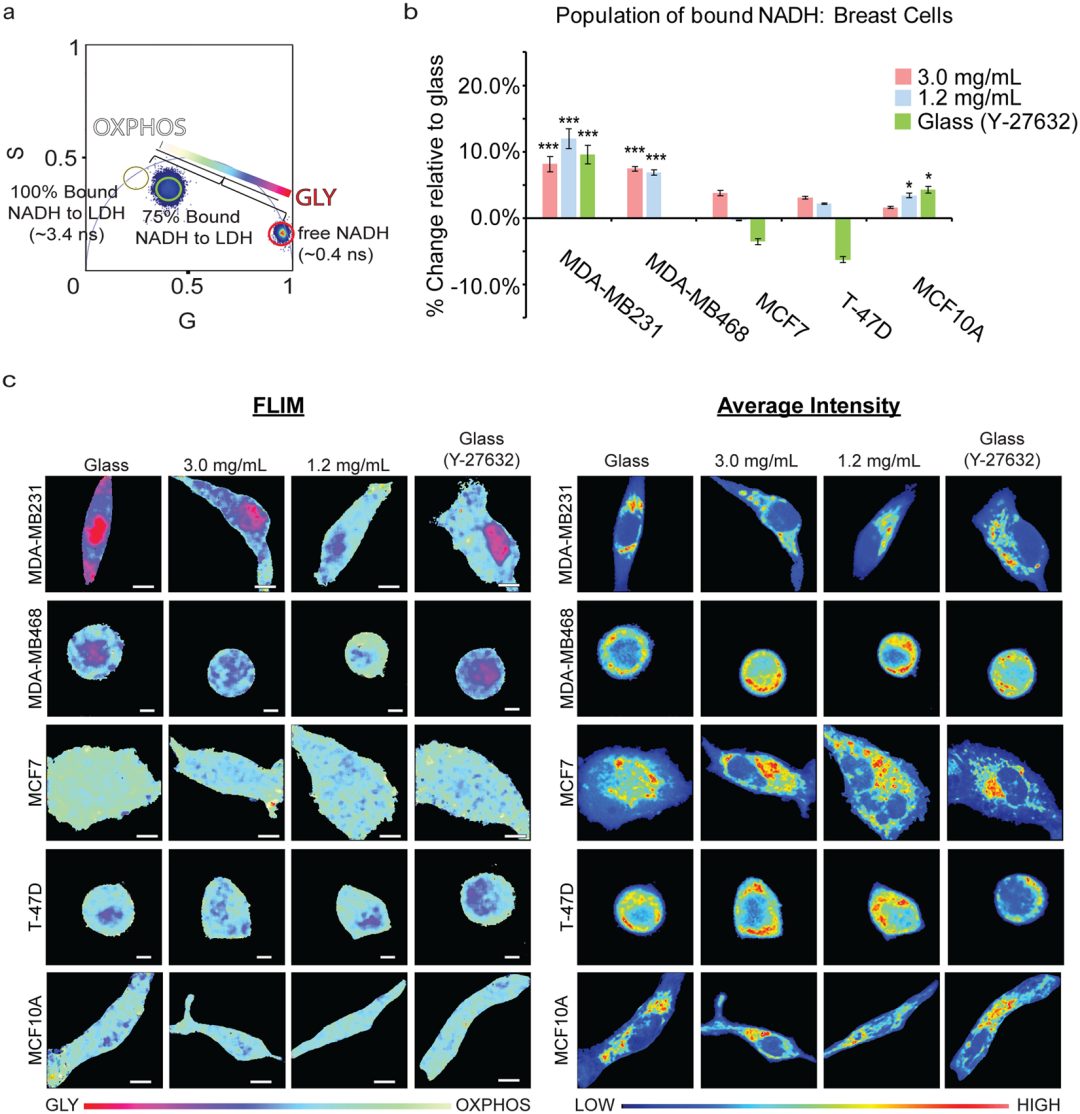
Metabolic indexes of MDA-MB231 (3.0 mg/mL: n = 71; 1.2 mg/mL: n = 53; Glass: n = 77; Glass (Y-27632): n = 33), MDA-MB468 (3.0 mg/mL: n = 20; 1.2 mg/mL: n = 20; Glass: n = 5; Glass (Y-27632): n = 5), MCF7 (3.0 mg/mL: n = 20; 1.2 mg/mL: n = 21; Glass: n = 20; Glass (Y-27632): n = 21), T-47D (3.0 mg/mL: n = 5; 1.2 mg/mL: n = 5; Glass: n = 5; Glass (Y-27632): n = 5), and MCF10A (3.0 mg/mL: n = 64; 1.2 mg/mL: n = 59; Glass: n = 63; Glass (Y-27632): n = 26) cells on various collagen densities. (**a**) An increased population of bound NADH to LDH (long lifetime NADH, cyan) is indicative of a more OXPHOS signature while an increased population of free NADH (short lifetime NADH, red) would indicate GLY. These two extremes create a linear “M-trajectory” where a mixed population of bound and free NADH, for example 75%, will lie between these two points. (**b**) Percent increase of bound NADH in MDA-MB231, MDA-MB468, MCF7, T-47D, and MCF10A cells relative to glass. (**c**) Colored images of FLIM of NADH and the average intensity of NADH within MDA-MB231, MDA-MB468, MCF7, T-47D, and MCF10A cells. n = total number of cells measured. *p < 0.05, **p < 0.01, and ***p < 0.001 by Student’s t-test. Scale bar: 5 µm. Error bars are based on standard deviation.

By measuring NADH fluorescence lifetimes with FLIM, we were able to non-invasively determine spatial shifts in the metabolism of different cell lines in response to collagen density. NADH has two different lifetimes when it is free in the cytosol, ~0.4 ns, or bound to a protein, ranging from ~1.4 ns to 9 ns^34–36^. Thus, we are able to distinguish the ratio of free and bound NADH at each pixel. For our studies, the lifetime of NADH bound to lactate dehydrogenase (LDH, ~3.4 ns) is used when quantifying the population of bound NADH, although there are many other possible enzymes^23,26^. The lifetime decay measured is Fourier transformed and displays a graphical representation of fluorescence lifetime on the phasor plot where all single exponential decay lifetimes are plotted on the semi-circle (called the universal circle) and all multi-exponential lifetimes are inside the semicircle representing the sum of linear combinations of single-exponential lifetimes. Figure 2a depicts the fluorescence lifetime of free NADH (0.4 ns) and 100% bound (3.4 ns) NADH to LDH. If the binding is not complete, we can calculate the ratio of bound to free NADH from the pure free and bound linear combination of lifetimes. In our control, the population of the percentage bound to NADH was 75%. This linear trajectory is indicative of GLY and OXPHOS state in live cells^28,37^.

Highly invasive MDA-MB231 cells were seeded on collagen and glass substrates to observe changes in free:bound ratios of NADH. In addition, the free:bound ratio of NADH in non-tumorigenic breast cells MCF10A were used as a control for a non-tumorigenic cell line. MDA-MB231 cells showed an 8.0% and 11.8% increase of bound NADH on 3.0 mg/mL and 1.2 mg/mL substrates, respectively, relative to those on glass (Fig. 2b). This indicates that as collagen density increased, the MDA-MB231 cells shifted from OXPHOS (white/cyan) to GLY (pink/red) (Fig. 2c). Similarly, MDA-MB-468 showed a 7.4% and 6.9% increase in bound NADH on the 3.0 mg/mL and 1.2 mg/mL substrates, respectively, marking a highly significant change (p < 0.001) when compared to the cells plated on glass. These results indicate that MDA-MB-468 cells shift to an increased OXPHOS signature when cultured on a less dense substrate. These cells on glass were then treated with 10 µM Y-27632, a ROCK inhibitor that decreases cell contractility, to assess if inhibiting the cell’s mechanosensing ability would shift the metabolism towards OXPHOS. Indeed, we see a significant change in the metabolic index (9.5% increase in bound NADH) in MDA-MB231 cells when Y-27632 was added (Fig. 2b). Similar results were seen on MDA-MB231 cells on glass when treated with 3.5 µM blebbistatin, showing a 16.1% increase relative to untreated glass samples (Supplementary Fig. S1). Interestingly, unlike the MDA-MB-231 cells, MDA-MB468 cells showed no significant difference in bound NADH compared to the cells plated on glass when treated with 10 μM of Y-27632. We also used both treatments on MDA-MB231 cells plated on 1.2 mg/mL and 3.0 mg/mL collagen and detected an increase in the bound NADH population as well (Supplementary Fig. S2). MCF10A cells also showed this shift in metabolism in response to collagen density but to a less drastic compared to the MDA-MB231 cells (Fig. 2b,c). MCF7 and T-74D cells also showed similar trends in the average increase of bound NADH relative to when they are grown on glass, although the changes were not significant. We also examined other cancer cell lines to determine if they also had the same response to collagen density. These results are shown below.

**Figure 3:**
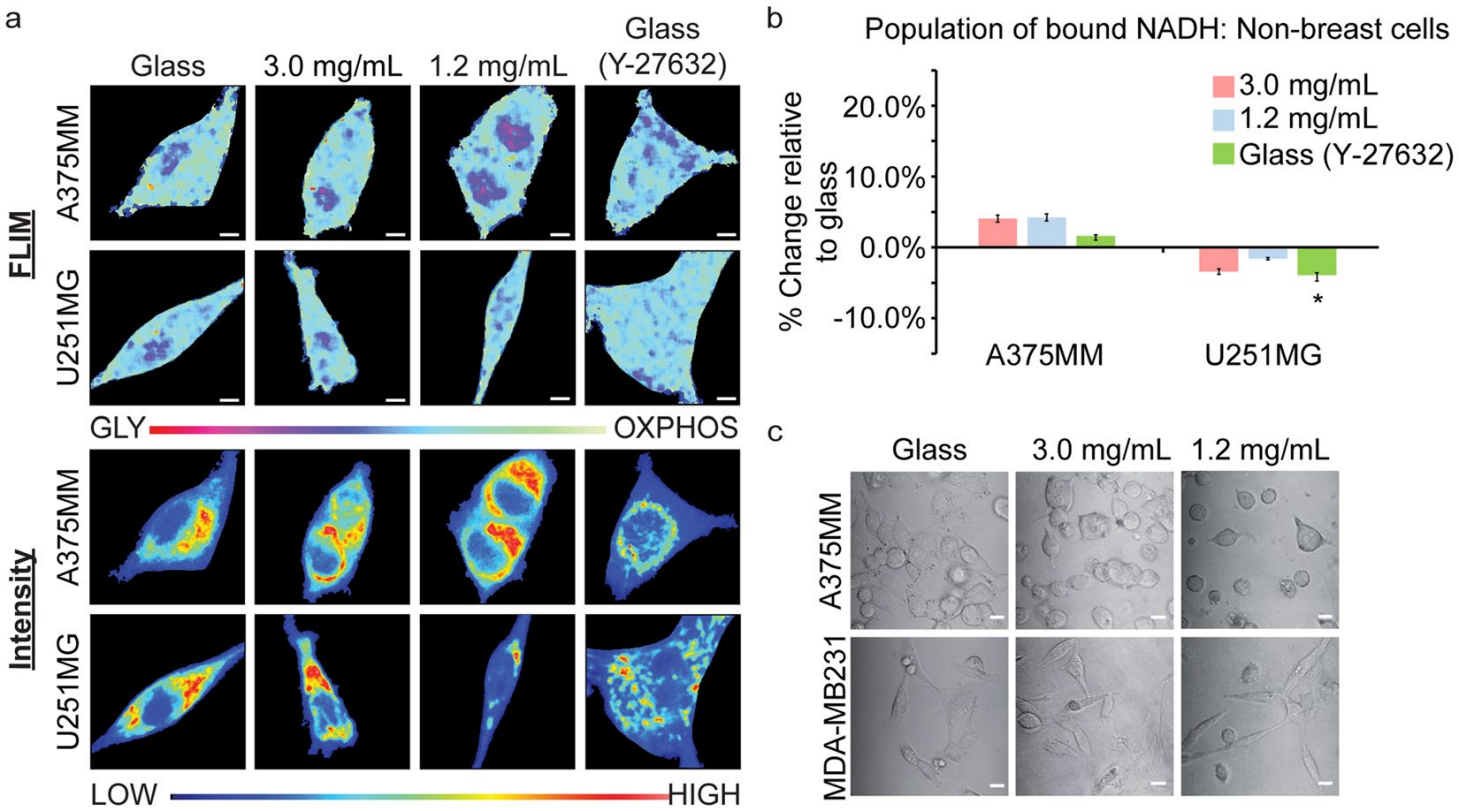
Metabolic indexes of A375MM (3.0 mg/mL: n = 24; 1.2 mg/mL: n = 23; Glass: n = 24; Glass (Y-27632): n = 28) and U251MG (3.0 mg/mL: n = 28; 1.2 mg/mL: n = 30; Glass: n = 26; Glass (Y-27632): n = 22) cells on various collage densities. (**a**) FLIM and average intensity images of NADH within A375MM melanoma and U251MG glioma cell lines. (**b**) Quantification of the percent change of NADH within A375MM and U251MG cell lines on various substrates relative to those on glass. (**c**) Transmitted optical images of A375MM and MDA-MB231 cells on different substrates. n = total number of cells measured. *p < 0.05, by Student’s t-test. Scale bar: 5 µm. Error bars are based on standard deviation.

Melanoma, A375MM, and glioblastoma, U251MG, cell lines showed different results than that of the TNBCs. A375MM cells showed no significant change in the free:bound ratio of NADH when on the 1.2 mg/mL or 3.0 mg/mL collagen substrates or glass (Fig. 3a,b). We noticed that these cells did not adhere as well on the collagen substrates due to their round morphology (Fig. 3c) which could be the reason why there was no change in metabolism as seen in the TNBCs. This further supports our hypothesis that the mechanosensing pathway plays an important role in cancer cell metabolism. The U251MG cells showed increased glycolytic signatures on average as collagen density decreased, although the results were not statistically significant. Previous studies have also shown that MDA-MB231 and U251MG have opposite trends of matrix degradation and invadopodia formation when they are cultured in different media supplemented with GLY or OXPHOS inhibitors^38^. This may explain our results, but further studies will need to be conducted to confirm them.

We isolated the metabolic phasor signature of the nucleus and the cytoplasm and compare them here across all surfaces in each cell line (Supplementary Fig. S3). Generally, the nucleus of the cell lines has a more GLY signature than the cytoplasm, but this did not significantly affect the results we found when looking at the entire cell within MDA-MB231 and MCF10A cells. However, within A375MM cells seeded on 3.0 mg/mL or 1.2 mg/mL collagen substrates, their populations of bound NADH were similar; but when looking at their nuclei and cytoplasmic, we began to see a separation between the two conditions, especially in the nuclei alone. The nuclei in A375MM cells on 3.0 mg/mL collagen substrates show a shift towards GLY where their population of bound NADH decreased by 29.5% compared to those on glass. U251MG cell nuclei metabolic indices were similar on all surfaces except for those on glass and treated with blebbistatin, which showed a 13.3% decrease in the population of bound NADH. We also looked at NIH3T3 fibroblast cells to observe if these changes can be seen in other cell types but the results showed no change (Supplementary Fig. S4).

The rest of this report will focus mainly on the MDA-MB231 and MCF10A cells to compare the results of cancerous and non-tumorigenic cell lines. In order to confirm that the fraction of free:bound ratio of NADH is modulated through collagen density, we conducted metabolism inhibition studies of each cell line when seeded on both collagen and glass substrates.

### Metabolism inhibition studies confirm that GLY and OXPHOS are modulated by collagen density

**Figure 4:**
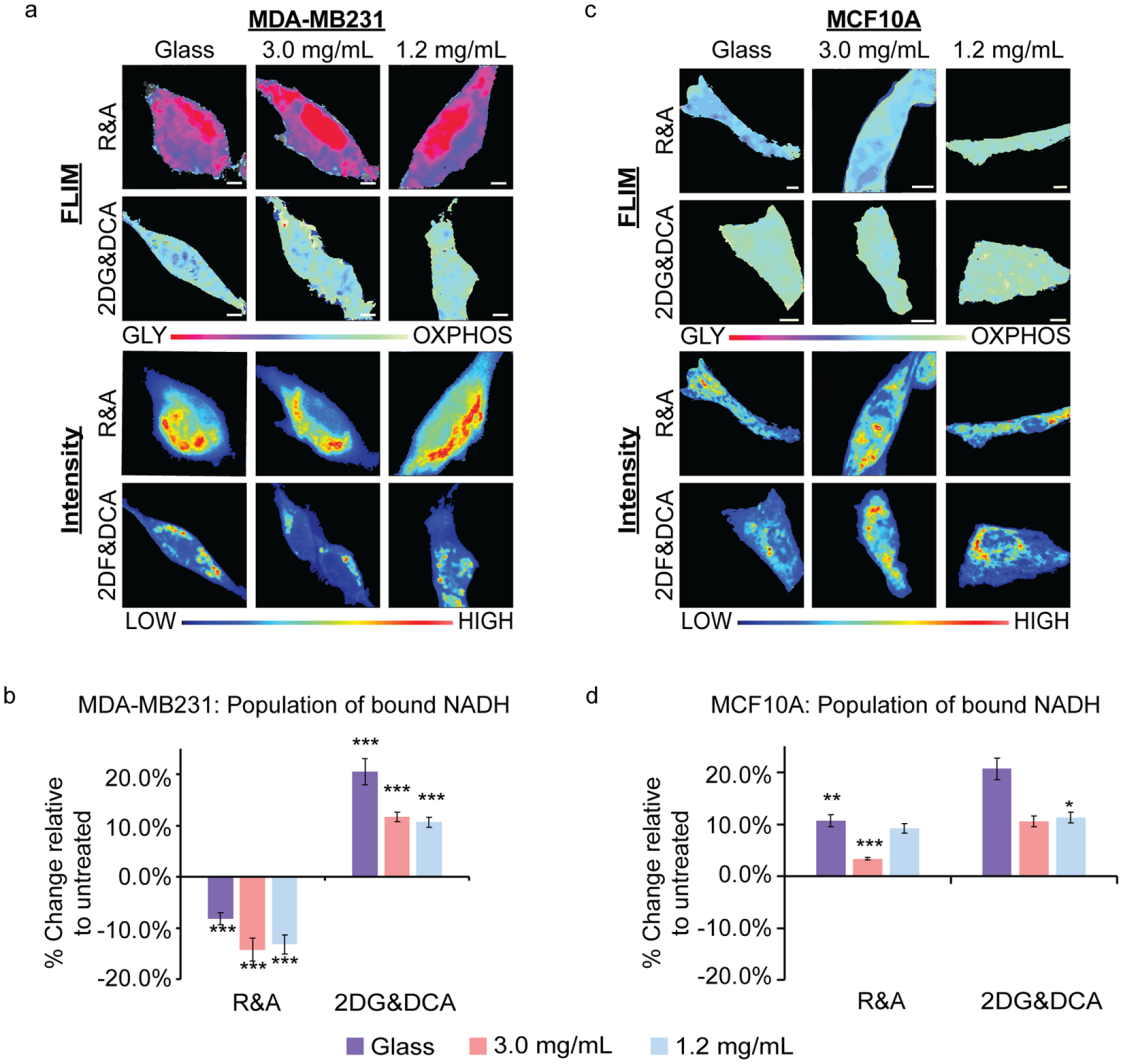
MDA-MB231 and MCF10A metabolic indexes when treated with metabolic inhibitors. (**a**) MDA-MB231 and (**b**) MCF10A cells treated with rotenone and antimycin A (R&A) or 2-deoxyglucose and dichloroacetate (2DG&DCA) for OXPHOS or GLY inhibition, respectively. FLIM images show the shifts in metabolic indexes with respective intensity images below. (**c**) Quantification of the change of bound NADH in MDA-MB231 with R&A (Glass: n = 29; 3.0 mg/mL: n = 29; 1.2 mg/mL: n = 29) or 2DG&DCA (Glass: n = 29; 3.0 mg/mL: n = 19; 1.2 mg/mL: n = 21) relative to untreated cells. (**d**) Quantification of the change of bound NADH in MCF10A with R&A (Glass: n = 25; 3.0 mg/mL: n = 20; 1.2 mg/mL: n = 15) or 2DG&DCA (Glass: n = 21; 3.0 mg/mL: n = 24; 1.2 mg/mL: n = 22) relative to untreated cells. n = total number of cells measured.*p < 0.05, **p < 0.01, and ***p < 0.001 or less by Student’s t-test. Scale bar: 5 µm. Error bars are based on standard deviation.

In order to ensure that the collagen density alters metabolism in MDA-MB231 and MCF10A cells and that these changes correlate with lifetime positions along the M trajectory, we treated these cell lines with oxidative phosphorylation and glycolysis inhibitors. We treated MDA-MB231 cells with 50 mM 2-deoxyglucose and 100 mM dichloroacetate (2DG&DCA) for GLY inhibition and 50 nM rotenone and 50 nM antimycin A (R&A) for OXPHOS. These inhibitors are known used to shift the metabolic signatures towards OXPHOS or GLY^4,27,30,39^. 2DG&DCA treatment showed an increased population of bound NADH of 20.5%, 11.7% and 10.6% for cells plated on glass, 3.0 mg/mL collagen, and 1.2 mg/mL collagen surfaces, respectively, relative to untreated cells (Fig. 4c). When MDA-MB231 cells were treated with R&A on glass, 3.0 mg/mL and 1.2 mg/mL collagen substrates, they showed significant decreases in the population of bound NADH, p < 0.0001, of 8.17%, 14.2% and 13.2%, respectively, relative to untreated cells. These treatments were also applied to MCF10A cells where we observed a significant decrease in the population of bound NADH only on 3.0 mg/mL collagen substrates, 3.39%, and glass, 10.7%, when treated with R&A (Fig. 4d). No changes were seen on the 1.2 mg/mL collagen substrates. However, there was a significant increase of 11.3% in these cells which were treated with 2DG&DCA.

## Discussion

The Warburg effect is the hallmark of cancer cell metabolism, described as an oncogene-directed glycolytic state even when oxygen is present^1,2,40^. This could be due to the high turnover of ATP production through glycolysis for energy production with glucose, although the alternative process of oxidative phosphorylation creates more product of energy per glucose molecule. Cancer cell invasion has also been shown to be modulated by changes in metabolism through changing cell media additives for energy consumption^38,41^ or the ECM stiffness and density^11,12,42–44^. However, the link between ECM stiffness or density and cell metabolic state (OXPHOS or GLY) is not clear. Recent studies that look at alterations of metabolism use invasive biochemical assays that do not report the spatial heterogeneous changes of metabolic response within cellular compartments, nor their cellular metabolic state. Our study used FLIM of NADH to measure real-time metabolic indexes of different cancer cell types in response to collagen density. This allows us to characterize which metabolic process occurs with pixel resolution in live cells. It is important to note that since different additives change cellular metabolism, our culture conditions for our cells remained the same through the study. Each cell line required different culturing conditions independently, but their media used was the same when measurements were taken. Thus, we are able to observe their respective metabolism changes in response to collagen density.

We have found that MDA-MB231, MDA-MB468, and MCF10A cells on substrates on two different collagen densities have an increasing population of free NADH, showing a more glycolytic signature of metabolism. The effect was even greater on the MDA-MB231 and MDA-MB468 TNBC metastatic cells. This correlates with previous studies in that breast cancer cells have increased migration and aggressiveness within denser collagen matrices^11,22,45^. In addition, cancer cells undergo aerobic glycolysis for energy production, thus increased collagen density could be a contributor to the metabolic shift towards glycolysis^1,39^. The ECM plays a key role in the cancer cell’s mechanosensing pathway through integrin signaling, and there is increasing evidence that this regulates cell migration and matrix degradation^9,33,38,46^. Increased ECM stiffness and density signals actin polymerization, upregulated integrin signaling, and stabilization of the focal adhesion complexes^18,19,47^. This increase of integrin signaling has been shown to also upregulate the PI3K/AKT/mTOR pathways and possible metabolism switching in cancer cells^4,20,21,48^. In addition, stimulating the actin-contractility of cells through external forces by shear flow or pulling of the cell membrane elevates glucose uptake^49,50^. Bays *et al*. has shown that this increased glucose uptake was also shown to increase ATP production for actin polymerization and stabilize E-cadherin contacts^49^. Our results expanded on these studies by looking at the specific metabolic indexes of cancer cells when introduced to various collagen density. We speculate that these same pathways are being activated in the TNBCs and are stimulated passively through focal adhesion-mediated interactions with the ECM.

When cells grown on glass substrates were treated with Y-27632 or blebbistatin, we inhibited their ability to undergo contraction through myosin-II and caused focal adhesion detachment from the substrate^51^. This, in turn, showed shifts in metabolic indexes from GLY to OXPHOS in MDA-MB231 and MCF10A cells. This confirms that it is through actin-mediated cell contractility that modulated these shifts in metabolism. Interestingly, the MDA-MB231 cells treated with Y-27632 had a similar NADH free: bound ratio as those grown on 3.0 mg/mL, which could mean that their degrees of contractility were similar to each the respective conditions. Those treated with blebbistatin had a much larger shift towards OXPHOS, surpassing the population of bound NADH of cells grown on 1.2 mg/mL collagen. Since blebbistatin directly affects myosin-II and is more potent than Y-27632, this was as expected. Previous studies have shown that stable adhesions have a developed mechanosensing network of actin, and these adhesions can grow in response to forces or increasing substrates stiffness^13,52–54^. Thus, cells on the denser substrates should have the most stable adhesions. In turn, they would also have increased FAK which promotes glucose consumption and plays a key role in the OXPHOS and GLY balance within cancer cells^55^.

The human melanoma cell lines, A375MM, in this study attached to 1.2 mg/mL and 3.0 mg/mL collagen substrates but did not spread as well as all other cells. This was an indication that their adhesions are not stable or favorable on these substrates^56–58^ and their mechanosensing ability could have been compromised and reduce mitochondria activity^59^. Consequently, this would fail to change the metabolic indexes of these cells as shown in our results; where the NADH free: bound of A375MM on 1.2 mg/mL or 3.0 mg/mL have no difference. This phenotype showed that there is a confirmed link between focal adhesion-mediated mechanosensing and cellular metabolism.

Glioblastoma cells, U251MG also showed no change in the the population of bound NADH as collagen density decreased. It is important to note that there are many other possible uses for pyruvate aside from OXPHOS in the mitochondria. Downstream of GLY are intermediates of the tricarboxylic acid cycle, such as citrate, which allows for the synthesis of lipids, proteins and nucleic acids, a demand for highly proliferating cells^60^. There is also fatty acid synthesis through citrate which is shown to correlate with the formation of invadopodia, which are actin-rich protrusions used for matrix invasion^39,41,43^. In addition, the pentose phosphate pathway could further increase the GLY and fraction of free NADH. All of the pathways mentioned could be elevated within MDA-MB231 cells, which could contribute to their difference in metabolic trends from the U251MG cells when collagen density is varied.

There is still the question of whether or not the shifts in metabolic signatures are unique to TNBCs. Based on the results reported here, this seems to be the case. Since we are changing the concentration of collagen to tune the density of the substrates, the density of ligands for integrin binding also varies. At the same time, an increase of substrate stiffness has been shown to upregulate FA signaling as well^61,62^. Thus, it is possible that the increase of integrin expression itself is the primary cause of the metabolism shifts^10,46^. Within *in vivo* systems, an increase in collagen density results in a stiffer ECM, which our system aimed to represent.

The TNBCs have a significant decrease in the fraction of bound NADH when plated on glass, 3.0 mg/mL and 1.2 mg/mL collagen, respectively. Although the percent of bound NADH of MDA-MB-468 cells on both collagen substrates increased compared to glass, there is no significant difference of bound NADH between the two collagen substrates. This variation from the MDA-MB-231 cell line could be due to the cell’s phenotype. MDA-MB-468 cells are much rounder than the MDA-MB-231 cells in every condition. This roundness likely indicates a decreased adherence to the substrate, and thus, when plated on the two much less dense collagen substrates, may have reached a plateau in its adhesion. This lack of change in adherence may be the cause of the non-significant changes in the free:bound ratio between the two collagen substrate conditions, however additional work is required to confirm this hypothesis. MCF7 and T-47D cells were shown to have similar trends of their average bound NADH when comparing them side-by-side. These two cell lines are similar in their genotype of ER+ , PR+ , and HER2-. Expression levels of ER+ , PR+ , HER2- are known to play an important role in cellular metabolism, thus these results are not surprising^63^.

We confirmed that the changes in the metabolic trajectory of the MDA-MB231 cells were reflective in cellular metabolism using the OXPHOS and GLY inhibitors. When these inhibitors were added, cells shifted their metabolism accordingly to their inhibitors but there were no significant metabolic differences across collagen densities within these changes (Supplementary Fig. S5a). However, the MCF10A cell lines did not show any changes in metabolic indexes across substrate densities in their untreated conditions. They did show substrate sensitivity only when OXPHOS was inhibited. When R&A was added to inhibit OXPHOS in MCF10A cells on the 3.0 mg/mL and glass substrates, there was a maximum decrease to around 63.8% of the population of bound NADH; however, those on 1.2 mg/mL collagen showed no significant change (Supplementary Fig. S5b). This could mean that on denser collagen substrates, these cells were more susceptible to metabolic changes when introduced to inhibitors. Additionally, this could also indicate that the metabolism of the MCF10A cells was behaving more like the MDA-MB231 cells on the denser matrices. When 2DG&DCA was added to inhibit GLY in MCF10A cells, we see an increase in the population of bound NADH to around 71.8% when grown on 1.2 mg/mL collagen substrate. Since OXPHOS and an environment with less collagen is preferable for the MCF10A cells, this could mean that this ECM provides an extra boost towards OXPHOS pathway when GLY is inhibited.

The phasor approach to FLIM of NADH allows isolation of the metabolic signature within sub-cellular compartments of the cells. Here, we focused on comparing the nuclei and cytoplasm of MDA-MB231, MCF10A, A375MM, and U251MG cell lines (Supplementary Fig. S3). We were able to see that the metabolic shifts within the nuclei and cytoplasm of MDA-MB231 and MCF10A cells are similar to their whole cell signature. However, within A375MM cells we were able to make distinctions of the population of bound NADH between surfaces, which were not detected when averaging over the entire cell. The nuclei of A375MM cells on 3.0 mg/mL collagen substrates has a significant decrease in the population of bound NADH with respect to those on glass. Thus, looking at the nuclear metabolic indexes can separate subtle changes that are hidden in whole cell readings. These distinctions seen could be due to nuclear processes, such as transcription or DNA repair, which has also been shown to affect the ratio of bound and free NADH^64,65^.

We have shown that focal adhesion-mediated contractility modulates cell metabolism in MDA-MB231 cancer cells. With the use of FLIM of NADH, we were able to non-invasively measure metabolic changes of cancer cell lines MDA-MB231, MDA-MB468, T-47D, MCF7, A375M, U251MG and within non- tumorigenic lines MCF10A, and NIH3T3. Particularly in triple-negative breast cancer lines, MDA-MB231 and MDA-MB468, we saw that less dense collagen substrates shifted cells to have a more OXPHOS metabolic signature due to their increased population of bound NADH. Further studies by inhibiting myosin-II contractility increased the population of bound NADH in MDA-MB231 cells across all surfaces and confirmed our hypothesis. This further supports that ECM mediated adhesions are upregulated due to substrate density and modulates metabolic signatures. We also confirmed that the changes in NADH free:bound in MDA-MB231 and MCF10A cells were due to GLY or OXPHOS by inhibiting these pathways with dichloroacetate and 2-deoxyglucose or rotenone and antimycin A, respectively. With our results combined with what is known in literature, there is a relationship between the mechanosensing and metabolism pathway in cancer cells and both play a critical role in regulating cancer invasiveness. This provides insight to develop therapies which target mechanosensing abilities of cancer cells to revert their metabolism similar to a more non-tumorigenic cell type or decrease their invasiveness.

## Materials and Methods

### Cell culturing and transfections

MDA-MB231, MDA-MB468, T-47D, MCF7, NIH3T3, and A375MM cells were cultured in Dulbecco’s Modified Eagle’s Medium (DMEM) with high glucose, L-glutamate, and sodium pyruvate (Genesee Scientific, San Diego, CA) supplemented with 10% heat-inactivated Fetal Bovine Serum (Thermofisher Scientific, USA) and 1% Penicillin-Streptomycin 100X Solution (Genesee Scientific, San Diego, CA). U251MG cells were cultured in DMEM/F12 with also with high glucose, sodium pyruvate and L-glutamine (Thermofisher Scientific, USA) supplemented with 10% heat-inactivated Fetal Bovine Serum (Thermofisher Scientific, USA) and 1% Penicillin-Streptomycin 100X Solution (Genesee Scientific, San Diego, CA). MCF10A cells were also cultured in DMEM/F12 with high glucose, sodium pyruvate and L-glutamine (Thermofisher Scientific, USA) supplemented with 5% horse serum (Thermofisher Scientific, USA), 20 ng/mL epidermal growth factor, 0.5 mg/mL Hydrocortisone (Sigma-Aldrich, St. Louis, MO), 100 ng/mL cholera toxin (Sigma-Aldrich, St. Louis, MO), 10 µg/mL insulin (Sigma-Aldrich, St. Louis, MO), and 1% Penicillin-Streptomycin 100X Solution (Genesee Scientific, San Diego, CA). All cell lines were incubated at 37 °C, 5% CO_2_.

### Collagen substrate monolayers

Substrates were made on 35 mm glass bottom imaging dishes which were treated for 5 minutes with UV-ozone. 1% v/v of 3-aminopropyltriethoxysilane (APTES) in deionized water were added and allowed to sit for 25 minutes at room temperature. The dishes were washed thoroughly with deionized water and brought to the biosafety hood for sterile handling. 1 mL of sterilized MilliQ water used to rinse the dish before collagen was added.

For collagen preparation, microcentrifuge tubes and reagents used were kept on ice for as long as possible while handling. Collagen I from rat tail (Corning, Corning, NY) was diluted with deionized water such that the final concentration was either 1.2 mg/mL or 3.0 mg/mL. 100 µL of 10X phosphate buffer saline (Thermofisher Scientific, USA) containing phenol red was added dropwise while vortexing as a pH indicator. 0.5 N NaOH was then added dropwise to the mixture with periodic vortexing until the solution became a slight pink (pH~7). The collagen was then added to the treated imaging dish (total volume of 1 mL) and incubated at 20 °C for 1 hour and then at 37 °C, 5% CO_2_ overnight. 0.25 × 10^6^ cells were then added to each dish the next day and then allowed to incubate at 37 °C, 5% CO_2_ overnight again before imaging took place.

### Inhibition studies

Contractility inhibition was done with Y-27632 (Selleckchem, US) or blebbistatin (Sigma-Aldrich, St. Louis, MO) at a working concentration of 10 µM and 3.5 µM, respectively. Each inhibitor was incubated for 10 minutes at room temperature before conducting FLIM/NADH imaging. MDA-MB231 or MCF10A cells were treated with sodium dichloroacetate (Sigma-Aldrich, St. Louis, MO) and 2-deoxyglucose (Sigma-Aldrich, St. Louis, MO) at a working concentration of 100 mM and 50 mM, respectively, for 6 hours at 37 °C, 5% CO_2_ to inhibit glycolysis^27^. Similarly, cells were treated for 10 minutes with 50 nM rotenone and 50 nM antimycin A at 37 °C, for oxidative phosphorylation inhibition studies before NADH lifetimes were measured.

### Characterization of collagen substrates

Rheology measurements of collagen substrates were conducted to obtain the storage (G′) and loss (G″) moduli. The collagen substrates were pre-made on 15 mm glass slides that were treated with UVO and APTES as described above. Collagen solutions of 1.2 mg/mL or 3.0 mg/mL were carefully pipetted onto the round glass slides and allowed to incubate at 20 °C for 1 hour and then at 37 °C overnight for 2 nights to mimic culturing conditions for imaging. The glass slides were placed on the stage of the AR-G2 rheometer (TA Instruments, New Castle, DE) that was kept at a constant temperature of 37 °C. A sand-blasted parallel plate geometry with a diameter of 25 mm was lowered to a gap distance of 0.3 mm so that it was in close contact with the surface of the collagen. Rheology measurements were conducted at a constant sinusoidal frequency of 1 Hz and 10% peak-to-peak strain and outputs of G’ and G” were recorded for a total of 10 minutes. A data point was taken every 60 seconds.

Second harmonic generation imaging of collagen substrates was conducted to characterize substrate density as previously^33,66^. Briefly, 2-photon excitation at 900 nm was used to generate second harmonics of collagen and collected with a bandpass filter at 460/80 nm with external photon-multiplier tubes (H7422P-40, Hamamatsu, Japan) and FastFLIM FLIMBox (ISS, Champaign, IL). 100 frames were collected and analyzed using image correlation spectroscopy on SimFCS (LFD, UCI). Spatial correlations were applied to each pixel at coordinate (*x*, *y*) of the complied SHG images with equation (1):

1 $${G}_{s}(\xi ,\phi )=\frac{ < I(x,y)I(x+\xi ,\,y+\phi { > }_{x,y}}{ < I(x,y){ > }_{x,y}^{2}}-1,$$

where *I* is the intensity. $\xi$ and $\phi$ are the spatial shifts in the *x* and *y* directions, respectively.

### Confocal and fluorescence lifetime imaging acquisition and analysis

FLIM images for MDA-MB231, U251MG, and A375MM cells were imaged on the Zeiss LSM 710 (Carl Zeiss, Jena, Germany), LSM 880 (Carl Zeiss, Jena, Germany), and Olympus Fluoview respectively. MCF10A, T-47D, and MDA-MB468 cells were also imaged on the LSM710. Metabolism inhibition studies of MDA-MB231 and MCF10A cells were imaged on the Olympus Fluoview. Images (256 × 256 pixel size) were taken at a pixel dwell time of 25.21 µs, 16.38 µs, and 20 µs for the LSM710, LSM880, and Fluoview, respectively. All microscope systems were coupled to with a two-photon Ti: Sapphire laser (Spectra-Physics MaiTai, Mountain View, CA) for NADH excitation at 740 nm with an Olympus 40X/0.8 NA water objective. The emission was separated at 690 nm in all systems followed by two bandpass filter at 460/80 nm and 540/50 nm with a with a dichroic mirror 495 nm long-pass filter. The signal was collected with an external photomultiplier tube (H7422P-40, Hamamatsu, Japan). A320 FastFLIM FLIMbox (ISS, Champaign, IL) was used to acquire the frequency domain of the lifetime of NADH until enough statistics were obtained. The experiments for all cells (except for the T47D and MDA-MB-468 cells) were repeated in triplicate. Images of coumarin-6 in ethanol were also taken as reference and calibration for FLIM measures across all microscopes.

SimFCS (LFD, UCI) was used to analyze the fluorescence lifetime of NADH at every pixel. The lifetime decay at each pixel was Fourier transformed and plotted on a phasor plot as previously described where each point on the phasor represents one pixel^37^. Each cell’s cluster of phasor points was averaged to obtain their S, G, and fraction bound value. Calculations for S and G for the in-phase and out-of-phase signals are shown in equations (2) and (3) respectively. *I* is the intensity of the pixel at point (x, y) and ω is the angular frequency of the light modulation. Average phasor plots of the data are shown in Supplementary Fig. S6.

2 $${S}_{x,y}=\frac{{\int }_{0}^{\infty }\,{I}_{x,y}(t)\sin (\omega t)dt}{{\int }_{0}^{\infty }\,{I}_{x,y}(z)\,dt}$$

3 $${G}_{x,y}=\frac{{\int }_{0}^{\infty }\,{I}_{x,y}(t)\cos (\omega t)dt}{{\int }_{0}^{\infty }\,{I}_{x,y}(t)\,dt}$$

The fraction of bound NADH is calculated based on the fact that any two points on the phasor plot (e.g. 100% free NADH and 100% bound NADH to LDH) can be connected by a line and any points along that line will be a linear representation of the two extremes. Thus, the experimental data will exist between 100% free NADH and 100% bound NADH, signifying samples that have a mixture of free and bound NADH. Those points that are closer to the phasor of bound NADH to LDH will have a higher population of bound NADH.

### Statistical analysis

Statistical significance was determined for experiments with the Student’s t-test (two-sample, variance based on F-test) in Microsoft Excel.

## Electronic supplementary material

Supplementary Material

## References

[1] Liberti MV, Locasale JW (2016). The Warburg Effect: How Does it Benefit Cancer Cells?. Trends Biochem. Sci..

[2] Vander Heiden MG, Cantley LC, Thompson CB (2009). Understanding the Warburg effect: the metabolic requirements of cell proliferation. Science.

[3] Warburg O, Wind F, Negelein E (1927). The Metabolism of Tumors in the Body. J. Gen. Physiol..

[4] Caino MC (2015). PI3K therapy reprograms mitochondrial trafficking to fuel tumor cell invasion. Proc. Natl. Acad. Sci. USA.

[5] Cunniff AB, Mckenzie AJ, Heintz NH, Alan K (2016). AMPK activity regulates trafficking of mitochondria to the leading edge during cell migration and matrix invasion Department of Pathology Department of Pharmacology University of Vermont Cancer Center University of Vermont, Burlington, VT 05405, USA Co. Mol. Biol. Cell.

[6] Zhao J (2013). Mitochondrial dynamics regulates migration and invasion of breast cancer cells. Oncogene.

[7] Desai SP, Bhatia SN, Toner M, Irimia D (2013). Mitochondrial localization and the persistent migration of epithelial cancer cells. Biophys. J..

[8] Estrella V (2013). Acidity generated by the tumor microenvironment drives local invasion. Cancer Res..

[9] Alexander NR (2008). Extracellular matrix rigidity promotes invadopodia activity. Curr. Biol..

[10] Artym VV (2015). Dense fibrillar collagen is a potent inducer of invadopodia via a specific signaling network. J. Cell Biol..

[11] Paszek MJ (2005). Tensional homeostasis and the malignant phenotype. Cancer Cell.

[12] Seewaldt V (2014). ECM stiffness paves the way for tumor cells. Nat. Med..

[13] Wells RG (2008). The role of matrix stiffness in regulating cell behavior. Hepatology.

[14] Liu Jaron, Wang Yilin, Goh Wah Ing, Goh Honzhen, Baird Michelle A., Ruehland Svenja, Teo Shijia, Bate Neil, Critchley David R., Davidson Michael W., Kanchanawong Pakorn (2015). Talin determines the nanoscale architecture of focal adhesions. Proceedings of the National Academy of Sciences.

[15] Kanchanawong P (2010). Nanoscale architecture of integrin-based cell adhesions. Nature.

[16] Bugyi B, Carlier M-F (2010). Control of actin filament treadmilling in cell motility. Annu. Rev. Biophys..

[17] Ponti A, Machacek M, Gupton SL, Waterman-Storer CM, Danuser G (2004). Two distinct actin networks drive the protrusion of migrating cells. Science.

[18] Hirata H, Tatsumi H, Lim CT, Sokabe M (2014). Force-dependent vinculin binding to talin in live cells: a crucial step in anchoring the actin cytoskeleton to focal adhesions. Am. J. Physiol. Cell Physiol..

[19] Gardel ML, Schneider IC, Aratyn-Schaus Y, Waterman CM (2010). Mechanical integration of actin and adhesion dynamics in cell migration. Annu. Rev. Cell Dev. Biol..

[20] Yang L (2015). Twist promotes reprogramming of glucose metabolism in breast cancer cells through PI3K/AKT and p53 signaling pathways. Oncotarget.

[21] Ata Rehman, Antonescu Costin (2017). Integrins and Cell Metabolism: An Intimate Relationship Impacting Cancer. International Journal of Molecular Sciences.

[22] Levental KR (2009). Matrix crosslinking forces tumor progression by enhancing integrin signaling. Cell.

[23] Ma N, Digman MA, Malacrida L, Gratton E (2016). Measurements of absolute concentrations of NADH in cells using the phasor FLIM method. Biomed. Opt. Express.

[24] Provenzano PP, Eliceiri KW, Keely PJ (2009). Multiphoton microscopy and fluorescence lifetime imaging microscopy (FLIM) to monitor metastasis and the tumor microenvironment. Clin. Exp. Metastasis.

[25] Bird DK (2005). Metabolic mapping of MCF10A human breast cells via multiphoton fluorescence lifetime imaging of the coenzyme NADH. Cancer Res..

[26] Datta R, Alfonso-García A, Cinco R, Gratton E (2015). Fluorescence lifetime imaging of endogenous biomarker of oxidative stress. Sci. Rep..

[27] Cinco R, Digman MA, Gratton E, Luderer U (2016). Spatial Characterization of Bioenergetics and Metabolism of Primordial to Preovulatory Follicles in Whole *Ex Vivo* Murine Ovary. Biol. Reprod..

[28] Stringari, C. *et al*. Metabolic trajectory of cellular differentiation in small intestine by Phasor Fluorescence Lifetime Microscopy of NADH. *Sci. Rep*. **2** (2012). 10.1038/srep00568 PMC3416911 22891156

[29] Sameni S, Syed A, Marsh JL, Digman MA (2016). The phasor-FLIM fingerprints reveal shifts from OXPHOS to enhanced glycolysis in Huntington Disease. Sci. Rep..

[30] Stringari C, Nourse JL, Flanagan LA, Gratton E (2012). Phasor Fluorescence Lifetime Microscopy of Free and Protein-Bound NADH Reveals Neural Stem Cell Differentiation Potential. PLoS One.

[31] Raub CB (2008). Image correlation spectroscopy of multiphoton images correlates with collagen mechanical properties. Biophys. J..

[32] Tilghman RW (2012). Matrix Rigidity Regulates Cancer Cell Growth by Modulating Cellular Metabolism and Protein Synthesis. PLoS One.

[33] Chiu C-L, Digman MA, Gratton E (2013). Cell matrix remodeling ability shown by image spatial correlation. J. Biophys..

[34] Skala MC (2007). *In vivo* multiphoton fluorescence lifetime imaging of protein-bound and free nicotinamide adenine dinucleotide in normal and precancerous epithelia. J. Biomed. Opt..

[35] Lakowicz JR, Szmacinski H, Nowaczyk K, Johnson ML (1992). Fluorescence lifetime imaging of free and protein-bound NADH. Proc. Natl. Acad. Sci. USA.

[36] Jameson DM, Thomas V, Zhou DM (1989). Time-resolved fluorescence studies on NADH bound to mitochondrial malate dehydrogenase. Biochim. Biophys. Acta.

[37] Digman MA, Caiolfa VR, Zamai M, Gratton E (2008). The phasor approach to fluorescence lifetime imaging analysis. Biophys. J..

[38] Van Horssen R (2013). Cancer cell metabolism regulates extracellular matrix degradation by invadopodia. Eur. J. Cell Biol..

[39] Morris BA (2016). Collagen Matrix Density Drives the Metabolic Shift in Breast Cancer Cells. EBioMedicine.

[40] Ward PS, Thompson CB (2012). Metabolic reprogramming: a cancer hallmark even Warburg did not anticipate. Cancer Cell.

[41] Scott KEN (2012). Metabolic regulation of invadopodia and invasion by acetyl-CoA carboxylase 1 and *de novo* lipogenesis. PLoS One.

[42] Artym VV, Zhang Y, Seillier-Moiseiwitsch F, Yamada KM, Mueller SC (2006). Dynamic interactions of cortactin and membrane type 1 matrix metalloproteinase at invadopodia: Defining the stages of invadopodia formation and function. Cancer Res..

[43] Gould CM, Courtneidge SA (2014). Regulation of invadopodia by the tumor microenvironment. Cell Adh. Migr..

[44] Provenzano PP (2008). Collagen density promotes mammary tumor initiation and progression. BMC Med..

[45] Haage A, Schneider IC (2014). Cellular contractility and extracellular matrix stiffness regulate matrix metalloproteinase activity in pancreatic cancer cells. FASEB J..

[46] Beaty BT (2013). β1 integrin regulates Arg to promote invadopodial maturation and matrix degradation. Mol. Biol. Cell.

[47] Ciobanasu C, Faivre B, Le Clainche C (2013). Integrating actin dynamics, mechanotransduction and integrin activation: the multiple functions of actin binding proteins in focal adhesions. Eur. J. Cell Biol..

[48] Lien, E. C., Lyssiotis, C. A. & Cantley, L. C. Metabolic Reprogramming by the PI3K-Akt-mTOR Pathway in Cancer. In *Recent results in cancer research. Fortschritte der Krebsforschung. Progres dans les recherches sur le cancer* **207**, 39–72 (Springer, Cham, 2016). 10.1007/978-3-319-42118-6_3 27557534

[49] Bays JL, Campbell HK, Heidema C, Sebbagh M, DeMali KA (2017). Linking E-cadherin mechanotransduction to cell metabolism through force-mediated activation of AMPK. Nat. Cell Biol..

[50] Hayashi T, Hirshman MF, Kurth EJ, Winder WW, Goodyear LJ (1998). Evidence for 5[prime] AMP-activated protein kinase mediation of the effect of muscle contraction on glucose transport. Diabetes.

[51] Martin K (2016). Spatio-temporal co-ordination of RhoA, Rac1 and Cdc42 activation during prototypical edge protrusion and retraction dynamics. Sci. Rep..

[52] Petit V, Thiery JP (2000). Focal adhesions: structure and dynamics. Biol. Cell.

[53] Kim DH, Wirtz D (2013). Focal adhesion size uniquely predicts cell migration. FASEB J..

[54] Bieling P (2016). Force Feedback Controls Motor Activity and Mechanical Properties of Self-Assembling Branched Actin Networks. Cell.

[55] Palorini R, Simonetto T, Cirulli C, Chiaradonna F (2013). Mitochondrial complex I inhibitors and forced oxidative phosphorylation synergize in inducing cancer cell death. Int. J. Cell Biol..

[56] Cavalcanti-Adam EA (2007). Cell spreading and focal adhesion dynamics are regulated by spacing of integrin ligands. Biophys. J..

[57] Massia SP, Hubbell JA (1991). Human endothelial cell interactions with surface-coupled adhesion peptides on a nonadhesive glass substrate and two polymeric biomaterials. J. Biomed. Mater. Res..

[58] Calderwood DA, Campbell ID, Critchley DR (2013). Talins and kindlins: partners in integrin-mediated adhesion. Nat. Rev. Mol. Cell Biol..

[59] Ochsner M, Textor M, Vogel V, Smith ML (2010). Dimensionality Controls Cytoskeleton Assembly and Metabolism of Fibroblast Cells in Response to Rigidity and Shape. PLoS One.

[60] DeBerardinis RJ, Lum JJ, Hatzivassiliou G, Thompson CB (2008). The Biology of Cancer: Metabolic Reprogramming Fuels Cell Growth and Proliferation. Cell Metab..

[61] Ng MR, Brugge JS (2009). A stiff blow from the stroma: collagen crosslinking drives tumor progression. Cancer Cell.

[62] Indra I, Beningo KA (2011). An *in vitro* correlation of metastatic capacity, substrate rigidity, and ECM composition. J. Cell. Biochem..

[63] Walsh A, Cook RS, Rexer B, Arteaga CL, Skala MC (2012). Optical imaging of metabolism in HER2 overexpressing breast cancer cells. Biomed. Opt. Express.

[64] Wright BK (2012). Phasor-FLIM analysis of NADH distribution and localization in the nucleus of live progenitor myoblast cells. Microsc. Res. Tech..

[65] Aguilar-Arnal L (2016). Spatial dynamics of SIRT1 and the subnuclear distribution of NADH species. Proc. Natl. Acad. Sci. USA.

[66] Raub CB (2007). Noninvasive assessment of collagen gel microstructure and mechanics using multiphoton microscopy. Biophys. J..

