# Supplementary material for: Collagen density modulates triple-negative breast cancer cell metabolism through adhesion-mediated contractility
Source: Sci Rep. 2018 Nov 20;8:17094. doi: 10.1038/s41598-018-35381-9 (PMC6244401; doi:10.1038/s41598-018-35381-9)
Supplement: Supplementary file 1 — Supplementary Material [file 41598_2018_35381_MOESM1_ESM.docx]

Collagen density modulates triple-negative breast cancer cell metabolism through adhesion-mediated contractility

Emma J. Mah^a,c^, Austin E. Y. T. Lefebvre^b,c^, Gabrielle E. McGahey^b,c^, Albert F. Yee^a,b^, and Michelle A. Digman^a,b,c*^

^a^ Department of Chemical Engineering and Materials Science, University of California, Irvine, Irvine, California, USA

^b^ Department of Biomedical Engineering, University of California, Irvine, Irvine, California, USA

^c^ Laboratory for Fluorescence Dynamics

*Correspondence should be sent to mdigman@uci.edu

# Supplementary Figures


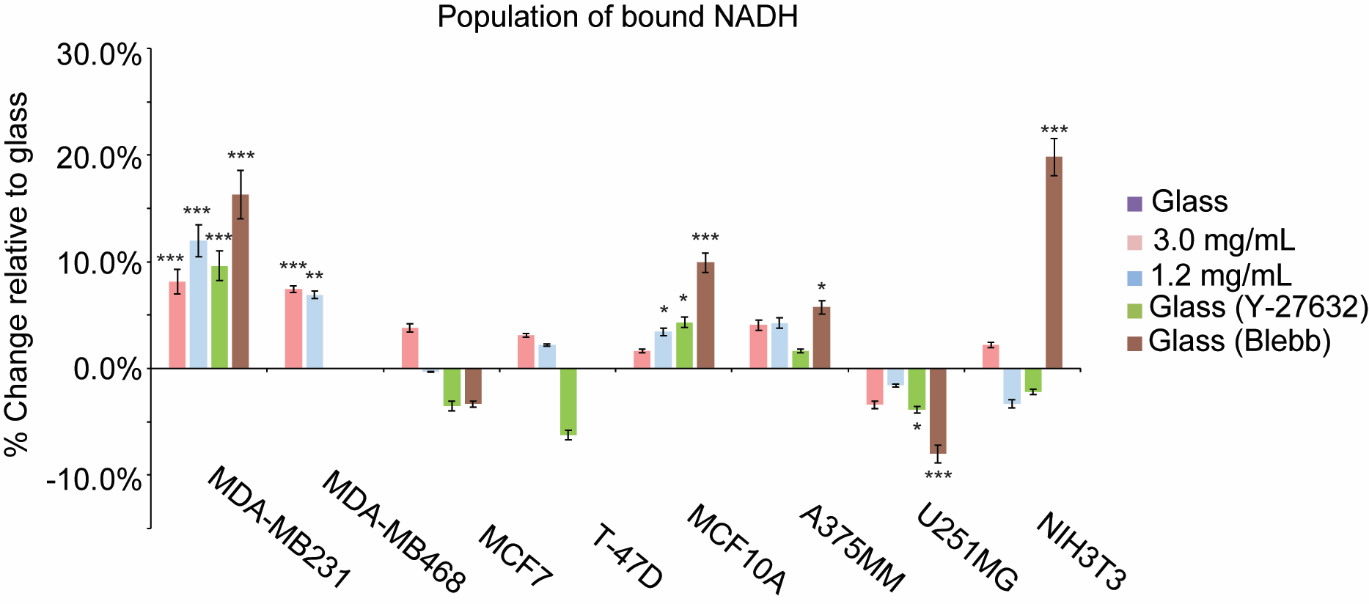


**Supplementary Figure S1:** Percent change in the population of bound NADH of MDA-MB231 (3.0 mg/mL: n=71; 1.2 mg/mL: n=53; Glass: n=77; Glass (Y-27632): n=33; Glass (Blebb): n=33), MDA-MB468 (3.0 mg/mL: n=5; 1.2 mg/mL: n=5; Glass: n=5; Glass (Y-27632): n=5), MCF7 (3.0 mg/mL: n=20; 1.2 mg/mL: n=21; Glass: n=20; Glass (Y-27632): n=21; Glass (Blebb): n=5), T-47D (3.0 mg/mL: n=5; 1.2 mg/mL: n=5; Glass: n=5; Glass (Y-27632): n=5), MCF10A (3.0 mg/mL: n=64; 1.2 mg/mL: n=59; Glass: n=63; Glass (Y-27632): n=26; Glass (Blebb): n=29), A375MM (3.0 mg/mL: n=24; 1.2 mg/mL: n=23; Glass: n=24; Glass (Y-27632): n=28; Glass (Blebb): n=31) and U251MG (3.0 mg/mL: n=28; 1.2 mg/mL: n=30; Glass: n=26; Glass (Y-27632): n=22; Glass (Blebb): n=31) and NIH3T3 (3.0 mg/mL: n=21; 1.2 mg/mL: n=15; Glass: n=20; Glass (Y-27632): n=19; Glass (Blebb): n=11) cells on collagen substrates and glass (treated with Y-27632 or blebbistatin, blebb) with respective to their glass conditions. n=total number of cells measured. *p<0.05, **p<0.01, and ***p<1e-3 or less by Student’s t-test. Error bars are based off of standard deviation.


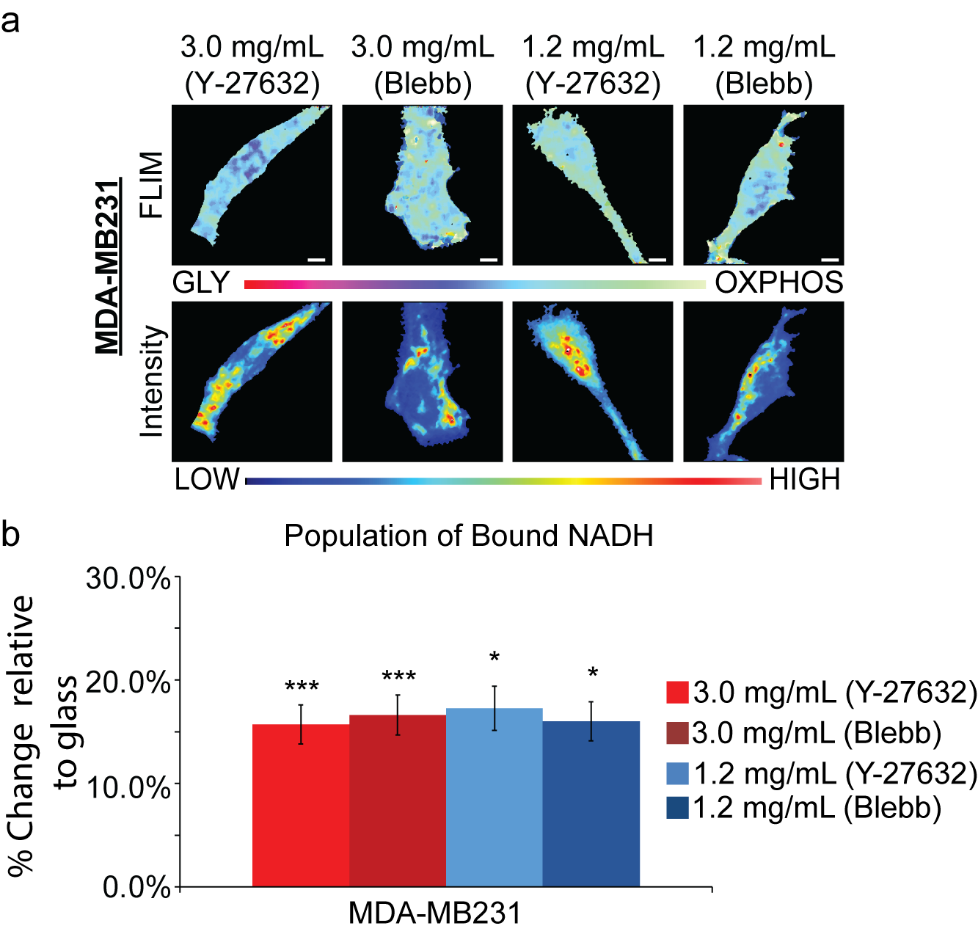


**Supplementary Figure S2:** MDA-MB231 metabolic indexes when treated with contractility inhibitors. (a) MDA-MB231 cells on 3.0 mg/mL and 1.2 mg/mL collagen treated with ROCK inhibitor Y-27632 or myosin-II inhibitor blebbistatin to prevent cell contractility. (b) Quantification of the percent change of the population of bound NADH in MDA-MB231 cells when treated with Y-27632 (3.0 mg/mL: n=7; 1.2 mg/mL: n=8) or blebbistatin (3.0 mg/mL: n=3; 1.2 mg/mL: n=4) relative to untreated conditions. n=total number of cells measured. *p<0.05 and **p<0.01 by Student’s t-test. Scale bar: 5 µm. Error bars are based off of standard deviation.


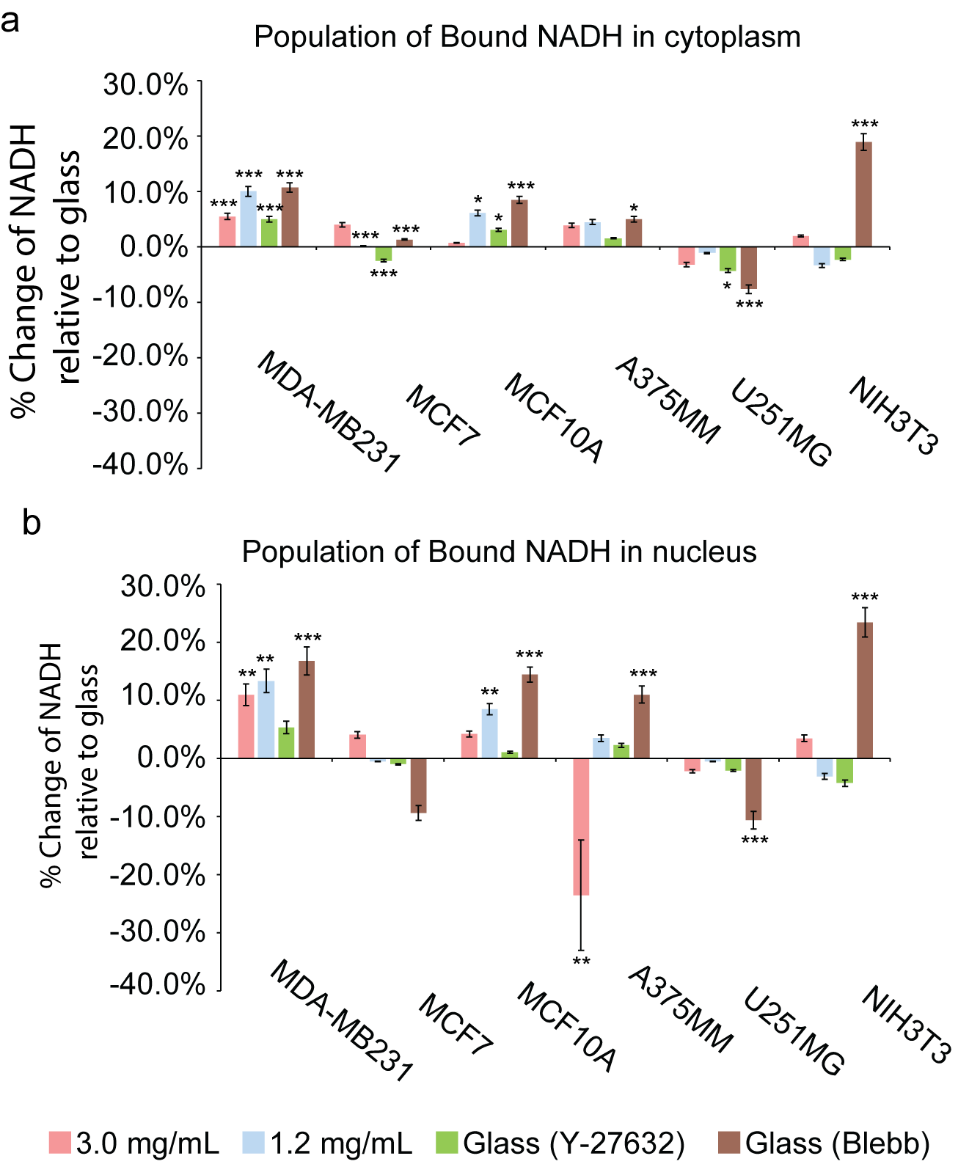


**Supplementary Figure S3:** NADH FLIM signatures of (a) cytoplasmic and (b) nuclear compartments within MDA-MB231, MCF7, MCF10A, A375MM and U251MG cells on varying substrates. * p<0.05, ** p<0.01, ***p<0.001 by Student’s t-test. Error bars are based off of standard deviation.


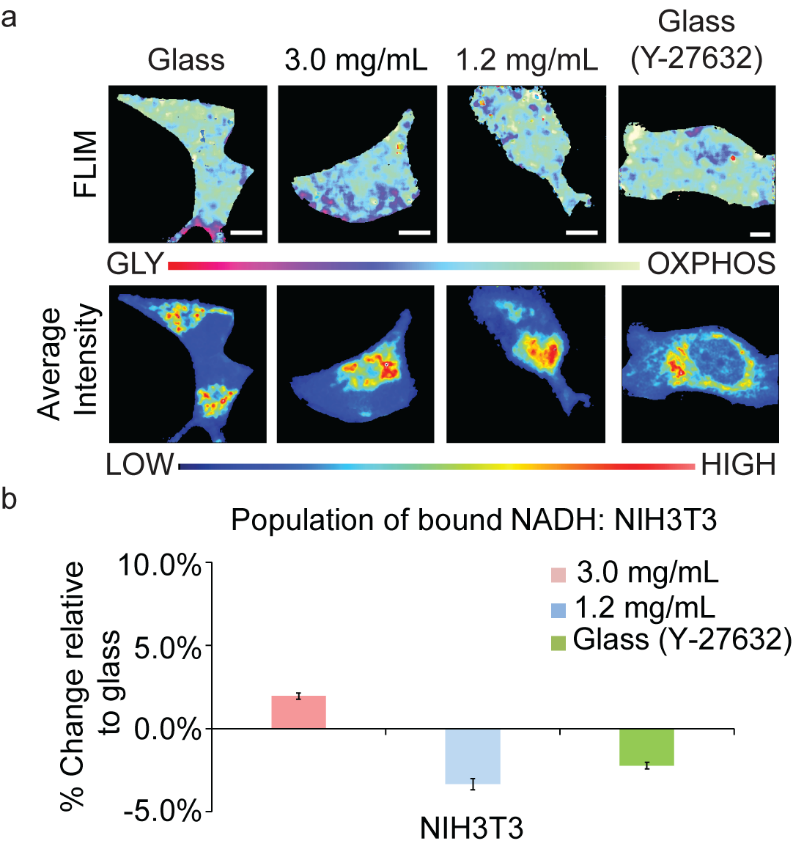


**Supplementary Figure S4:** Metabolic indexes of NIH3T3 cells (3.0 mg/mL: n=21; 1.2 mg/mL: n=15; Glass: n=20; Glass (Y-27632): n=10). n=total number of cells measured. (a) FLIM and average intensity images of NADH within NIH3T3 fibroblast cell lines. (b) Quantification of the percent change of NADH within NIH3T3 cell on various substrates relative to those on glass.


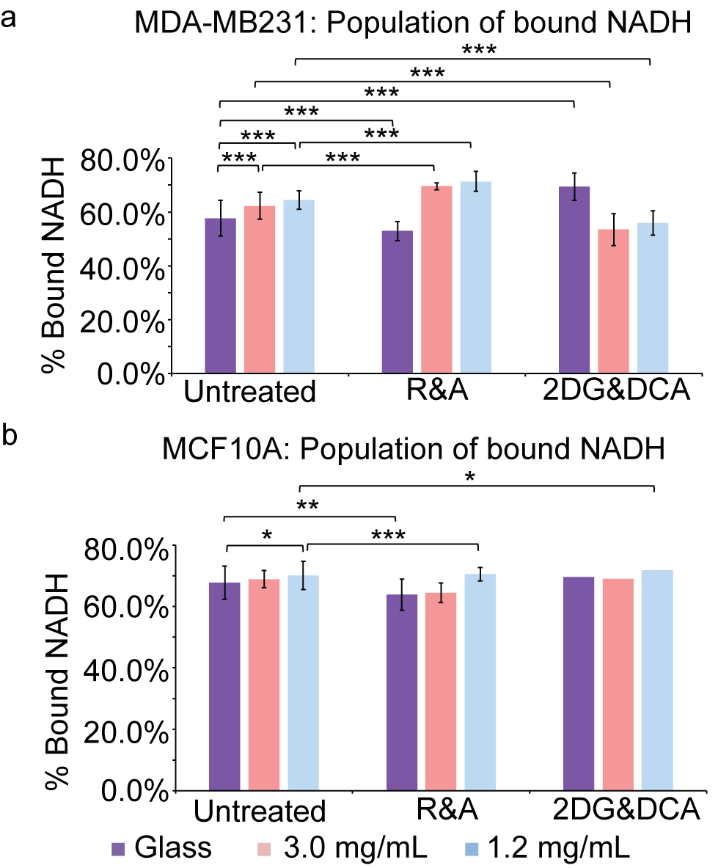


**Supplementary Figure S5:** (a) Raw percent bound of NADH of MDA-MB231 and (b) MCF10A cells on glass, 1.2 and 3.0 mg/mL collagen substrates when treated with R&A, 2DG&DCA and untreated. * p<0.05 and **p<0.01, ***p<0.001 by Student’s t-test.


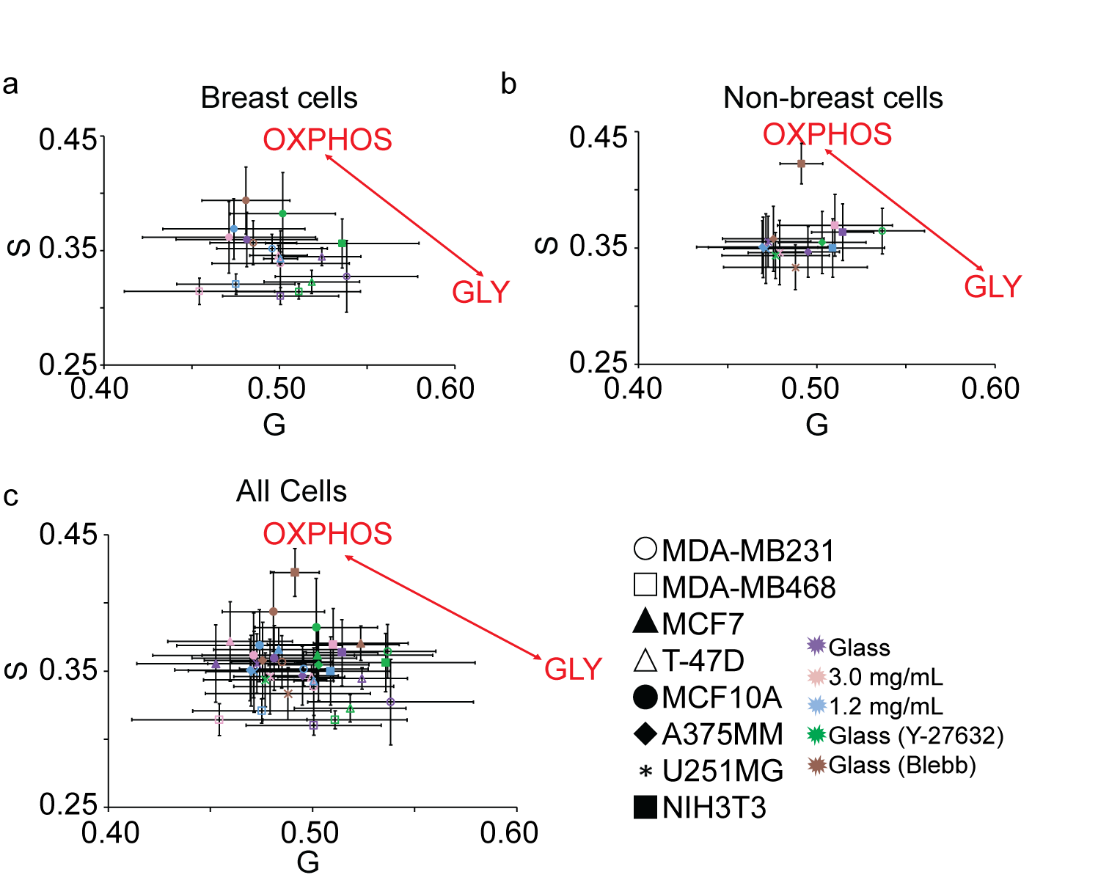


**Supplementary Figure S6:** Phasor plots of all cells. (a) Breast cells’ average whole cell phasor plots of their metabolic indexes when seeded on glass, 1.2 mg/mL, and 3.0 mg/mL collagen substrates. Untreated and treated conditions with blebbistatin or Y-27632 are shown. (b) Non-breast cells’ average whole cell phasors of their metabolic indexes when seeded on glass (untreated and treated with blebbistatin or Y-27632), 1.2 mg/mL, and 3.0 mg/mL collagen substrates. (c) Combined average whole cell phasors from (a) and (b). Error bars are based off of standard deviation.
